# LiDAR-Only Crop Navigation for Symmetrical Robot

**DOI:** 10.3390/s22228918

**Published:** 2022-11-18

**Authors:** Rémy Guyonneau, Franck Mercier, Gabriel Freitas Oliveira

**Affiliations:** LARIS (Laboratoire Angevin de Recherche en Ingénierie des Systèmes), SFR MATHSTIC, University of Angers, F-49000 Angers, France

**Keywords:** crop navigation, LiDAR measurements, line extraction, fuzzy controller, ROS/Gazebo

## Abstract

This paper presents a navigation approach for autonomous agricultural robots based on LiDAR data. This navigation approach is divided into two parts: a line finding algorithm and a control algorithm. The paper proposes several line finding algorithms (based on PEARL/Ruby approach) that extract lines from a LiDAR data set. Once the lines have been processed from the data set, a control algorithm filters these lines and, using a fuzzy controller, generates the wheel speed commands to move the robot among the crop rows. This navigation approach was tested using a simulator built on ROS middle-ware and Gazebo (the source codes of the simulation are available on GitHub). The results of the simulated experiments show that the proposed approach performs well for a large range of crop configurations (with or without considering weeds, with or without holes in the crop rows…).

## 1. Introduction

### 1.1. Context

The development of robotic tools for agriculture is a growing field. Current research is exploring a variety of tasks ranging from weeding robots [1,2] to harvesting robots [3,4]. Most of the platforms being developed aim to be autonomous.

One of the most fundamental problems for autonomous robots is autonomous navigation. In an agricultural application, such as market gardening, the robot must be able to follow the crop rows regardless of the weather conditions, the surrounding luminosity, or the configuration of the field.

Most recent works consider GPS or camera data for navigation [5,6]. However, these sensors do not provide reliable data in all conditions. Indeed, at the edge of a forest or in a greenhouse, the GPS signal is degraded. In these situations, range sensors are commonly used. This paper proposes an approach based on LiDAR (Light Detection and Ranging) data.

**Figure 1:**
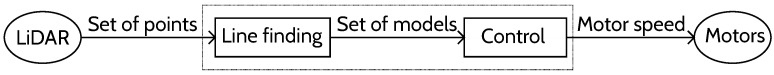
Overview of the approach: the LiDAR sensor provides points, and, from those points, lines (models) are extracted and from those lines the motor speeds are deduced.

**Figure 2:**
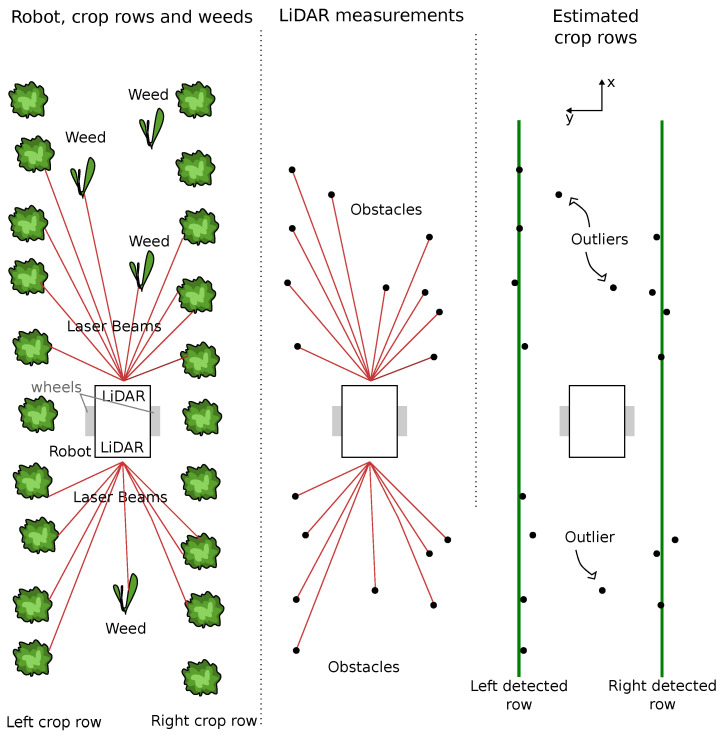
The line finding problem: The objective is to identify the crop rows from LiDAR data. The left part of the figure depicts a top view of the scene (a robot moving between two crop rows), the middle part depicts the data from the sensors (i.e., points corresponding to plants) and the right part shows an ideal result from the line finding algorithm (that is, the left and right crop rows have been identified from the LiDARs data set).

The work presented here aims to propose an approach for the navigation of a symmetrical robot based on LiDAR sensor data only. This paper presents a number of novelties, including:

- The improvement of the existing line finding algorithm (Figure 1 and Figure 2 introduces the line finding idea). The interested reader can refer to Section 4 for the algorithm behavior comparison;
- The setup of filters and a fuzzy controller to have a full navigation stack. The control algorithm presented in this paper (Section 3) allows the simulated robot to move autonomously through the entire field. Considering a symmetrical robot eases the row changing maneuver;
- The development of a new simulation based on ROS and Gazebo. To ease the reusability of the work and to be able to test the algorithms for several crop configurations, a simulation has been developed based on ROS Robot Operating System middle-ware and Gazebo (Section 4). Note that all the simulator source codes and documentation are available in Ref. [7].

### 1.2. Overview of the Approach

Using LiDAR data, without prior knowledge of the field, the objective is for the robot to be able to autonomously navigate between the rows. The considered robot is a symmetrical two-wheeled differential robot. The navigation approach presented in this paper can be divided into two parts: a line finding algorithm and a control algorithm, as depicted in Figure 1.

To be able to navigate among the crops, it is necessary to identify the crop rows from the LiDAR data. This step corresponds to the line finding process of the approach and is depicted in Figure 2. One should note that it is assumed that the robot is equipped with 2D LiDAR sensors and that the sensors can detect the crop plants and the weeds (i.e., the LiDAR is low enough or the plants are high enough). In other words, plants have to be detected by the sensors, otherwise this approach can not be applied. Nomenclature resumes the considered notations.

The line finding algorithm thus provides a set of models (i.e., a set of lines) that best fit the points detected by the sensors. Those models (lines) are then filtered in order to hopefully correspond to actual rows in the crop. This filtering is done by the control algorithm, which also provides a fuzzy controller to maintain the robot in the middle of the rows.

Section 2.1 presents several line finding algorithms while Section 3 describes the considered control algorithm. Section 4 presents the designed simulation and the results obtained when testing the algorithms. Finally, Section 5 concludes this paper.

## 2. Materials and Methods

### 2.1. Line Finding Algorithms

To ease the reading of this paper, the Ruby algorithm introduced in Ref. [8] is presented in Section 2.1.1. The novelties brought to this algorithm are then presented in Section 2.2.

#### 2.1.1. The Ruby Algorithm

The Ruby algorithm is based on the Pearl method presented in [9]. Pearl, thus Ruby, is a method that aims at minimizing a function called energy. In the latter, a model $L_{j}:f_{j}\left(x\right)=a_{j}x+b_{j}$ corresponds to a line, and $L_{i}$ depicts a set of models. A point *p* (an obstacle detected by the LiDARs) is associated with a model L(p). Note that L(p) could be the empty model $L_{\emptyset }$ (if *p* is an outlier for instance). These notations are listed in Nomenclature.

The considered energy function is described in Equation (1).

(1) $$E\left(L\right)=E_{\emptyset }\left(L\right)+E_{N}\left(L\right)+\sum_{L_{j}\in L\setminus \left\{L_{\emptyset }\right\}}E_{L}\left(L_{j}\right).$$

For a set of models L, the energy E(L) is divided into three terms: the outlier energy $E_{\emptyset }\left(L\right)$, Equation (2), the penalty energy $E_{N}\left(L\right)$, Equation (3), and the sum of all the model energies $E_{L}\left(L_{j}\right)$, Equation (6).

The outlier energy $E_{\emptyset }\left(L_{i}\right)$ aims at taking into account the points that are not associated with a model (i.e., those that are associated with the empty model $L_{\emptyset }$). It is defined as

(2) $$E_{\emptyset }\left(L_{i}\right)=\rho \cdot \sum_{p\in P(L_{\emptyset })}1,$$

where $\sum_{p\in P(L_{\emptyset })}1$ is the number of points in the current empty model $L_{\emptyset }\in L_{i}$ and ρ is a constant value that is heuristically chosen. As it is assumed that the LiDAR will detect more crops than weeds, it is appropriate to penalize the points that are not attached to any model (i.e., outliers and weeds).

The penalty function tends to favor the association of two nearby points (according to the Euclidean distance) to the same model. This energy is defined as

(3) $$E_{N}\left(L_{i}\right)=\lambda \cdot \sum_{(p,q)\in N}w_{pq}\cdot \delta_{\neq }\left(L\left(p\right),L\left(q\right)\right),$$

where N is the set of neighboring points such that an element (p,q)∈N corresponds to two points *p* and *q* in the same neighborhood, with *p* associated with the model L(p) and *q* associated with the model L(q). λ is a constant heuristically chosen. $\delta_{\neq }\left(L\left(p\right),L\left(q\right)\right)$ is defined as

(4) $$\delta_{\neq }\left(L\left(p\right),L\left(q\right)\right)\left\{\begin{array}{cc} 1 & \mathrm{if}\;L(p)\neq L(q)\\ 0 & \mathrm{otherwise} \end{array}\right.,$$

additionally

(5) $$w_{pq}=\exp\frac{-||p-{q||}^{2}}{\zeta^{2}},$$

where ||p−q|| is the Euclidean distance between the points *p* and *q*, while ζ is a heuristically chosen constant.

Finally, the energy of a model $L_{j}$ is defined as

(6) $$E_{L}\left(L_{j}\right)=\sum_{p\in P(L_{j})}||p-L_{j}\left|\right|,$$

where $||p-L_{j}\left|\right|$ is the Euclidean distance between a point *p* attached to the model $L_{j}$ and that model.

#### 2.1.2. Details of the Algorithm

**Figure 3:**
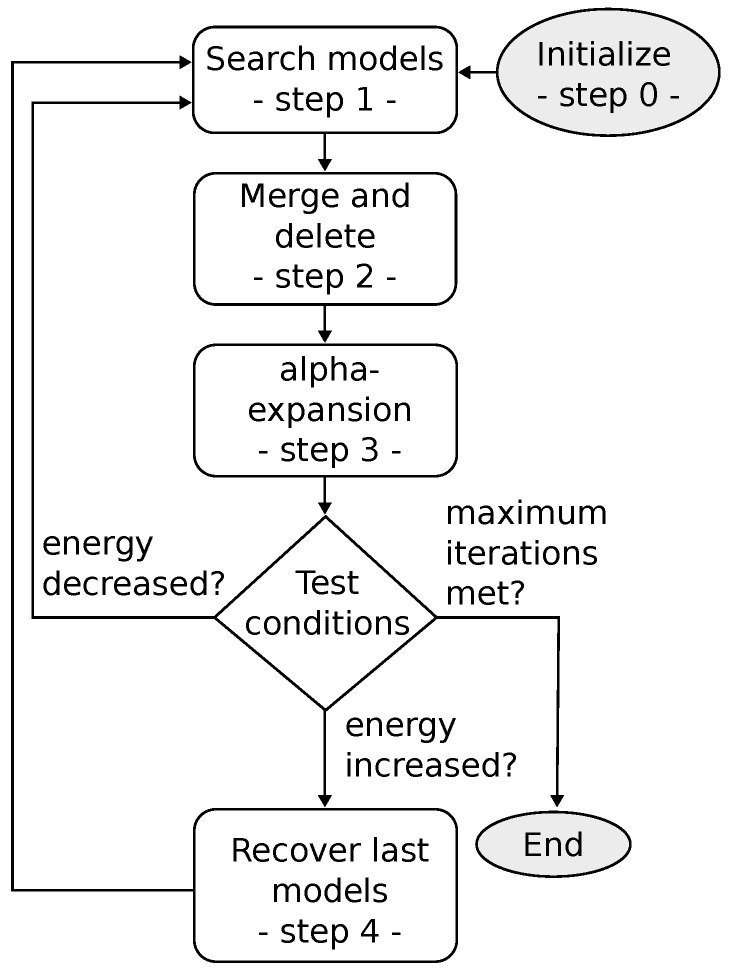
Overview of the Ruby method.

The Ruby algorithm is presented in Figure 3 and detailed in Algorithm 1. The different steps are explained in the latter.

1. Line 1 Algorithm 1, which corresponds to the initialization. The initial set of models $L_{0}$ is initialized with the one from the previous find line computation $L_{i}^{-1}$. Note that if it is the first ever call of line finding, the initial set of models is initialized with only an empty model: $L_{i}^{-1}=\left\{L_{\emptyset }\right\}$ with $P(L_{\emptyset })=Z$, i.e., all the points are associated with the empty model, Z being the set of all the points. This is relevant because in a crop navigation context, it is assumed that between two LiDAR scans, the robot will sense mostly the same crops and weeds: the rows will not be completely different from two consecutive find line calls.
2. Lines 5 to 10 Algorithm 1. Three random points (*p*, *q*, *z*) are picked in $L_{\emptyset }$ and the best model is computed (linear regression) so that it fits those points. This creates a new model that is added to the model set. This process is then repeated until half of the outliers are attached to a model (or below a threshold).
3. Lines 11 to 16 Algorithm 1, the merging part. It merges all the models that are too close to each other.
4. Lines 17 to 20 Algorithm 1, the deleting part. It removes all the models that do not meet the constraint
  (7) $$\frac{E_{L}\left(L_{j}\right)}{\psi_{L_{i}}\left(L_{j}\right)\cdot \sum_{p\in P(L_{j})}1}-\alpha <0,$$
  where $E_{L}\left(L_{j}\right)$ is the energy of the model $L_{j}$ as described in Equation (6), α is the constant chosen heuristically and $\psi_{L}\left(L_{j}\right)$ is the number of models of $L_{i}$ parallel to the model $L_{j}$. $\psi_{L_{i}}\left(L_{j}\right)$ is defined as
  (8) $$\psi_{L_{i}}\left(L_{j}\right)=\sum_{L_{k}\in L_{i}\setminus \left\{L_{j},L_{\emptyset }\right\}}\delta_{}\left(L_{j},L_{k}\right),$$
  with
  (9) $$\delta_{}\left(L_{j},L_{k}\right)\left\{\begin{array}{cc} 1 & \mathrm{if}\;|a_{j}-a_{k}|-\beta <0\\ 0 & \mathrm{otherwise} \end{array},\right.$$
  where $a_{j}$ and $a_{k}$ are the slopes of the models $L_{j}$ and $L_{k}$, and β a constant chosen heuristically.
5. Lines 21 to 24 Algorithm 1. All the points are detached from their models and re-attached to the closest model. Then the new energy is computed for the complete set of models.
6. Lines 26 to 27 Algorithm 1. If the energy of the new set of models is worse (bigger) than the previous iteration, the previous set of models is kept instead of the new computed one.

**Algorithm 1:** Ruby algorithm.

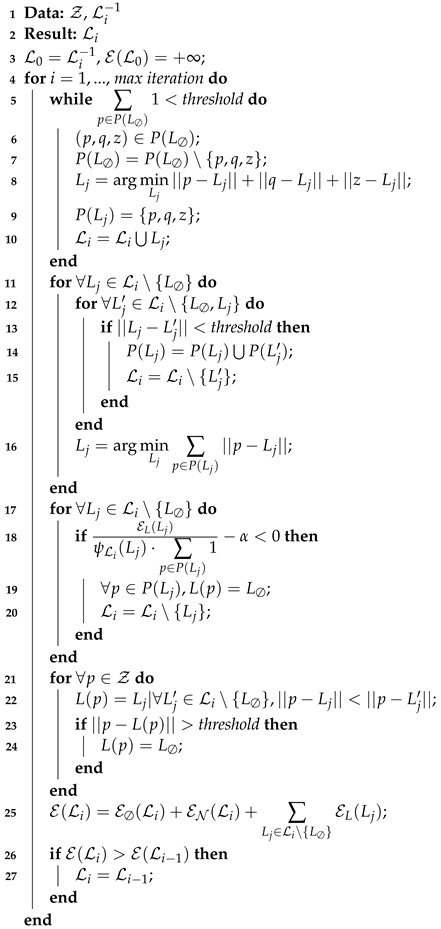



### 2.2. Ruby Suggested Refinements

In this section we will present refinements for the Ruby algorithm. These refinements are part of the originality of the work presented in this paper. They lead to an improvement of the robot navigation results, as shown in Section 4.

#### 2.2.1. Ruby Genetic

Pearl and Ruby algorithms are very similar to genetic algorithms [10]. To enhance this resemblance, the Ruby genetic refinement proposes to modify the deleting part of step 2 (Figure 3). In the classical Ruby approach, a constant α is used (Equation (7)) to decide if a model has to be removed or not. Ruby genetic proposes a fitness criterion instead, named $\varphi_{j}$ and defined as

(10) $$\varphi_{j}=\frac{b_{j}^{2}+\tau \cdot E_{L}\left(L_{j}\right)}{\psi_{L_{i}}\left(L_{j}\right)\cdot \sum_{p\in P(L_{j})}1},$$

with $E_{L}\left(L_{j}\right)$, defined in Equation (6), $\psi_{L_{i}}\left(L_{j}\right)$m defined in Equation (8) and where τ is a heuristically chosen constant. By using this criterion, the best models are sorted, while the others are removed.

#### 2.2.2. Ruby Genetic One Point

The idea of this refinement is based on the assumption that one plant of the crop will generate several LiDAR readings. Thus, the data that are close to each other can be merged together as they may belong to the same plant. This leads to the computation of a new point set named $Z^{*}$. The process is described in Algorithm 2.

For this Ruby Genetic One Point refinement, the input data is no longer Z, as defined in Algorithm 1, but $Z^{*}$. The rest of the algorithm remains the same as for the Ruby genetic.
**Algorithm 2:** The computation of $Z^{*}$.
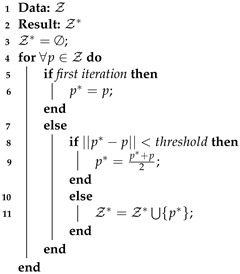


#### 2.2.3. Ruby Genetic One Point Positive/Negative

This refinement proposes to change the way models are found (step 1 in Figure 3). For the classical Ruby approach, three points are randomly taken from the empty model $L_{\emptyset }$. However, assuming that the robot will most of the time be parallel to the crop rows, and not perpendicular to them, the points can be separated into two sets $P_{left}\left(L_{\emptyset }\right)$ and $P_{right}\left(L_{\emptyset }\right)$, defined as

(11) $$\begin{array}{c} P_{left}\left(L_{\emptyset }\right)=\left\{p\in P\left(L_{\emptyset }\right)\right\}|y_{p}<0, \end{array}$$

(12) $$\begin{array}{c} P_{right}\left(L_{\emptyset }\right)=\left\{p\in P\left(L_{\emptyset }\right)\right\}|y_{p}>0. \end{array}$$

**Figure 4:**
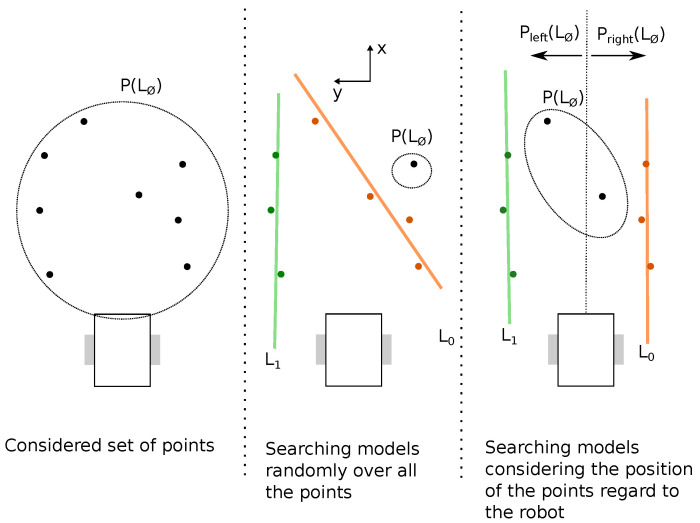
The idea behind considering $P_{left}\left(L_{\emptyset }\right)$ and $P_{right}\left(L_{\emptyset }\right)$. Without doing so, there is a chance that the model research considers improbable models regarding the orientation of the robot (middle part of the illustration), reducing the chance of finding the *best models*. On the other hand, while dividing the model research into left and right points, as the rows should be on the left or on the right, the chances of finding the correct models are increased (the right section of the illustration).

Then the research for models is done into those two subsets. Thus, step 1 of the Ruby algorithm (Figure 3), i.e., lines 5 to 10 of Algorithm 1, becomes as detailed in Algorithm 3. An example of this new model research approach is depicted in Figure 4.
**Algorithm 3:** Ruby Genetic One Point Positive/Negative-Search models-step 1.
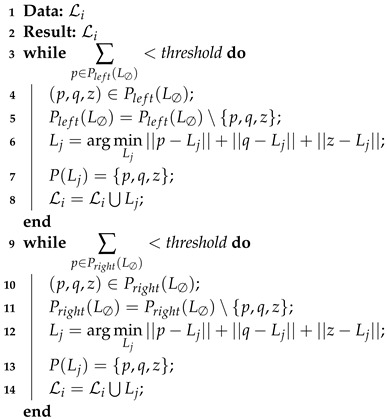


#### 2.2.4. Ruby Genetic One Point Positive/Negative Infinity

The last considered refinement was proposed after noticing that as a point can only be associated with one model, a *bad model* can consume a point of a potentially *good model*, as it can be noticed in the middle part of Figure 4. To handle this limitation, this refinement proposes to allow a point to be attached to several models at the same time.

The main drawback of this approach is that it is time-consuming: all the points have to be checked all the time.

## 3. The Control Algorithm

**Figure 5:**
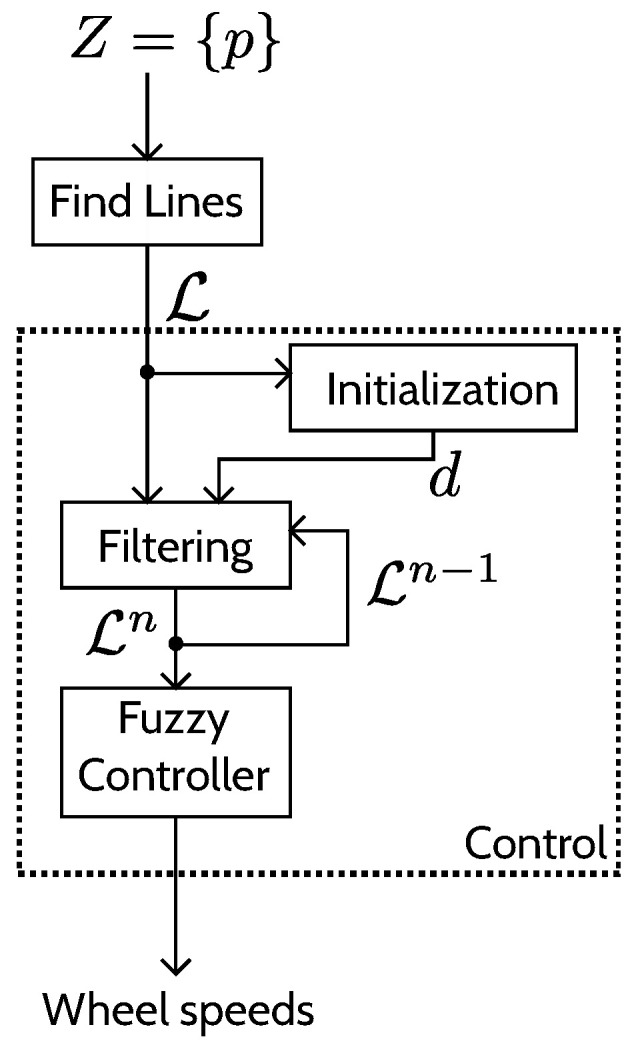
Details of the control approach.

Once models (lines) have been extracted from the points (the data set from the LiDAR), a control can be processed based on those models. The control algorithm presented here can be divided into three main steps: an initialization, filtering, and control step. The steps are depicted in Figure 5.

### 3.1. Initialization

The idea of the overall approach is to allow the robot to autonomously navigate into a crop with the minimum of prior information (width and length of the rows for instance). The only assumptions about the field are that:

- The field is organized in straight lines, as this is the case most of the time [11,12];
- The distance between two rows does not change inside a crop;
- The initial position of the robot is somewhere between two crop rows;
- The initial orientation of the robot is roughly parallel with the crop rows.

It can be noticed that the two first assumptions (about the field configuration) do not reduce the application as it is done in most crop row detection approaches [13,14]. The last two assumptions (about the robot’s initial position) may be more restricting, but as long as it is assumed that the robot can be placed in the crop, it is not an issue.

Based on these assumptions, an initialization step has been developed. The idea is to compute the distance between the rows from the models given by the line finding algorithm before starting to move the robot.

**Figure 6:**
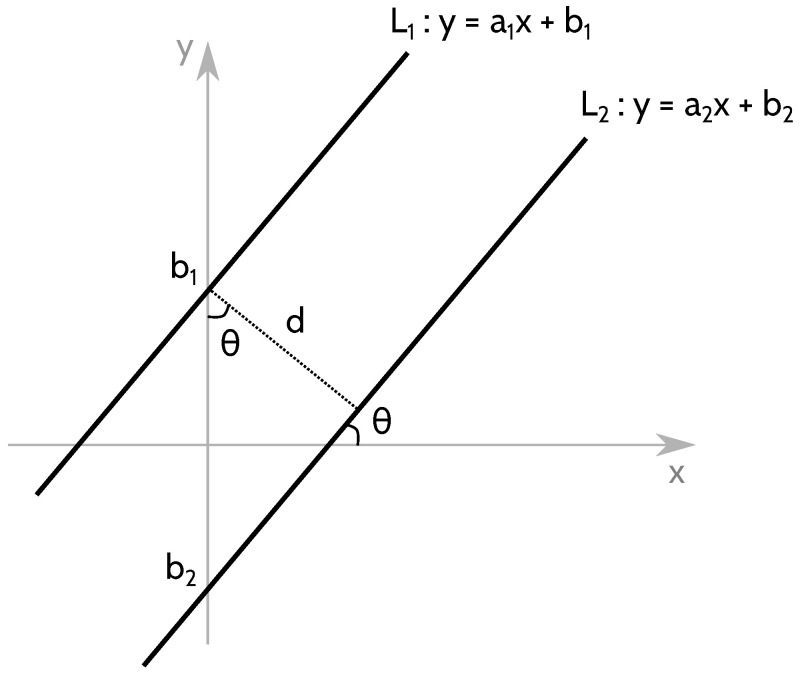
Computing the distance between two parallel models $L_{1}$ and $L_{2}$. Note that $a_{1}=a_{2}$ for the models to be parallel.

To that end, the initialization process extracts from the models the closest pair of models that are equidistant to the robot (the robot should be in the middle of two rows for its initial position) and have a slope close to 0 (the robot should be parallel to the crop rows). Once a pair of models $\left\{L_{1},L_{2}\right\}$ is found, the distance between the rows is computed as follows (see Figure 6).

$$\begin{array}{c} \cos\left(\theta_{r}\right)=\frac{d}{|b_{1}-b_{2}|},\\ d=\cos\left(\theta_{r}\right)\cdot \left|b_{1}-b_{2}\right|,\\ d=\cos\left(\arctan\left(a_{1}\right)\right)\cdot \left|b_{1}-b_{2}\right|,\\ d=\cos\left(\arctan\left(a_{2}\right)\right)\cdot \left|b_{1}-b_{2}\right|, \end{array}$$

With $\theta_{r}$ the orientation of the robot, *d* the distance between two rows in the field and $\left(a_{i},b_{i}\right)$ the parameters of the model $L_{i}$. As

(13) $$\cos\left(\arctan\left(a\right)\right)=\frac{1}{\sqrt{a^{2}+1}}$$

It can be concluded that

(14) $$d=\frac{|b_{1}-b_{2}|}{\sqrt{a_{1}^{2}+1}}=\frac{|b_{1}-b_{2}|}{\sqrt{a_{2}^{2}+1}}$$

To have a more stable distance, this distance computation is done several times (according to several results of the line finding algorithm) and an average of all the computed distances is done to get an estimation of the distance between two rows. This estimation is then stored for use by the robot’s navigation.

### 3.2. Filtering

**Figure 7:**
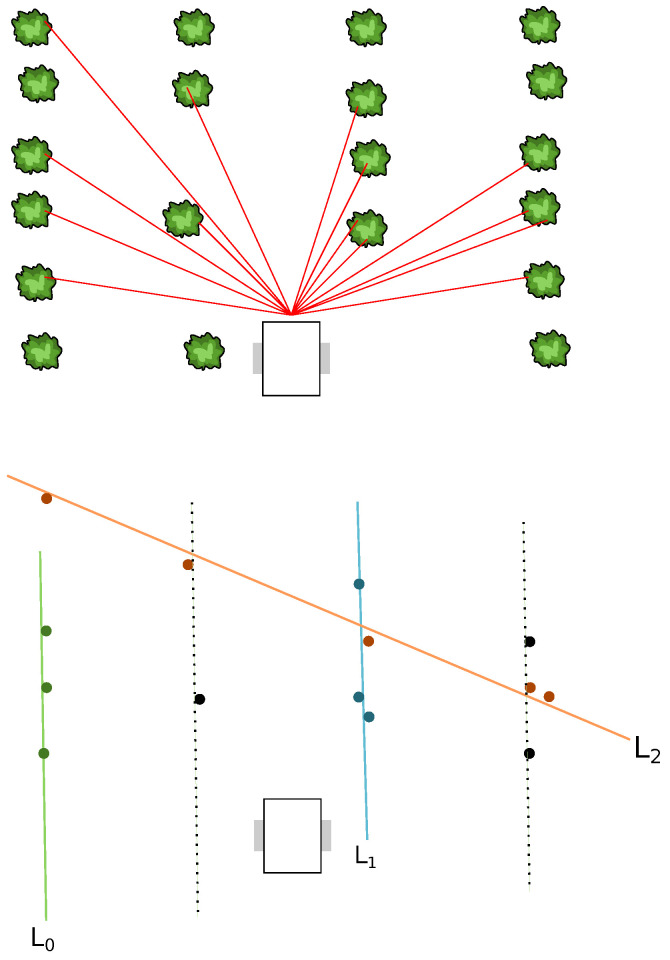
Assuming that from the top configuration, the line finding algorithm returns the models $L_{0}$, $L_{1}$, and $L_{2}$ (bottom part of the figure). The expected behavior of the filter is to keep the models $L_{0}$ and $L_{1}$ (removing the model $L_{2}$) and knowing the distance *d* between the rows, to compute the missing models (dotted lines). Note that for the bottom part of the figure, the colors of the dots correspond to the models the points are associated to.

The line finding algorithm, as detailed in Section 2.1, provides models (lines) according to a point set (LiDAR data). However, most of the time some returned models do not correspond to effective rows in the field (Figure 7). Filtering is then required to remove models that do not meet the field geometry assumptions (distance *d* between the rows and previously computed and verified models). This filter is presented in Algorithm 4 and is detailed in the latter.

The required data are:

- L: the set of models provided by the line finding algorithm;
- $L^{n,-1}=\left\{L^{1,-1},L^{2,-1},\cdots ,L^{i,-1},\cdots ,L^{n,-1}\right\}$: the filtered model set of the previous iteration with *n* a defined number of models that is constant during the robot navigation. The *i* index represents the position of the row identified by the model (from left to right).
- *d*: the distance between the rows, computed during the initialization step (Section 3.1).

**Algorithm 4:** Model filtering.
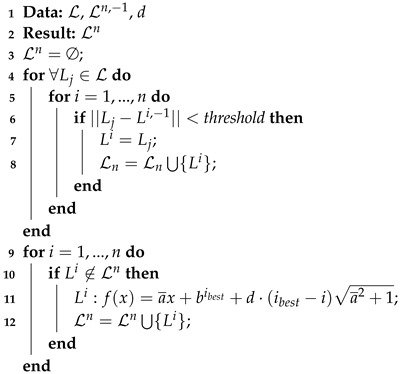


**Figure 8:**
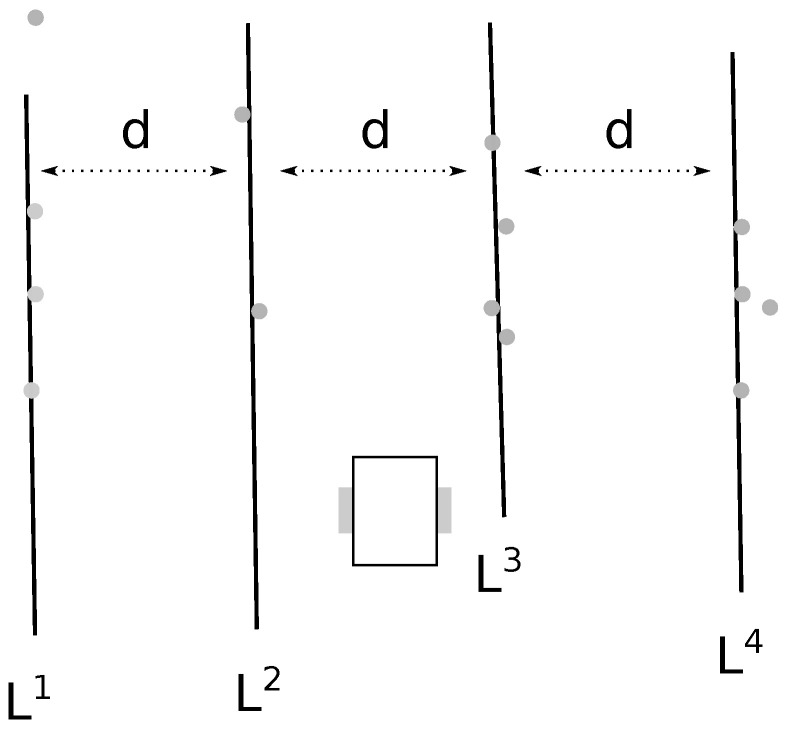
The filtered models $L^{n}$, with n=4, resulting in the configuration depicted in Figure 7. The models $L^{2}$ and $L^{4}$ are computed according to $L_{0}$, $L_{1}$, and *d*.

The result of the filtering is a filtered model set $L^{n}=\left\{L^{1},\cdots ,L^{i},\cdots ,L^{n}\right\}$, with *n* a fixed number of models. Note that a filtered model $L^{i}$ may be extracted from the models L provided by the line finding algorithm or may be computed according to other filtered models $L^{j}$ and the row distance *d*. Figure 8 depicts an expected filtered result according to the situation described in Figure 7.

The filtering algorithm can be divided into two parts: searching for expected models and computing missing models.

The search for the expected models, lines 4 to 8 of Algorithm 4, is based on the assumption that between two iterations the robot will not move significantly, i.e., the rows are mostly at the same place as they were for the prior iteration. It means that the new models should be similar to the previous ones. Thus, from the new model set L provided by the line finding algorithm, we search all the models that are close enough (according to a threshold) to the previous filtered ones $L^{n,-1}$. If a match is found, this new model is added to the new filtered set $L^{n}$. Note that if during this step no match is found, the previous filtered models are kept ($L^{n}=L^{n,-1}$). If this happens for several iterations then the robot is considered lost and stops navigating.

Once all the models from L have been tested, some expected filtered models $L^{i}$ may not have a match in the model set L (at most n−1). That is, they have to be computed regarding the found filtered models and the row distance *d*, lines 9 to 12 of Algorithm 4. The computation of a missing model $L^{i}$ is done as

(15) $$L^{i}:f\left(x\right)=\bar{a}x+b^{i_{best}}+d\cdot \left(i_{best}-i\right)\cdot \sqrt{{\bar{a}}^{2}+1},$$

where $\bar{a}$ is the average slope of the found filtered models

(16) $$\bar{a}=\frac{\sum_{L^{i}\in L^{n}}a^{i}}{\sum_{L^{i}\in L^{n}}},$$

and where $i_{best}$ is the index of the best match

(17) $$i_{best}=\arg\min_{i}\left|\right|L^{i}-L^{i,-1}\left|\right|.$$

At the end of this filter step we have *n* models $L^{n}=\left\{L^{1},\ldots ,L^{n}\right\}$ that should be consistent with the crop and the robot configuration. These are the models that are considered by the controller presented in the next section.

### 3.3. Fuzzy Controller

A fuzzy controller is a classical approach when dealing with a mobile robot control that can be applied to agricultural robots [12,15]. The objective of the controller detailed in the latter is, according to the filtered models $L^{n}$, to compute the needed wheel speeds for the robot to move between the rows. The fuzzy controller presented here has three steps:

- Fuzzification: transforms the inputs into fuzzy inputs (Section 3.3.1);
- Rule evaluation: defines how the inputs impact the outputs (Section 3.3.2);
- Defuzzification: from the fuzzy outputs, generated by the fuzzy inputs and the rules, defines a non-fuzzy output (Section 3.3.3).

#### 3.3.1. Inputs of the Controller

From the filtered models $L^{n}$ we are only interested in the model $L^{left}$ that is directly to the left of the robot and the model $L^{right}$ that is directly to the right of the robot. For instance, in the example depicted in Figure 8, $L^{left}=L^{2}$ and $L^{right}=L^{3}$.

The orientation of the robot in the field is computed as:

(18) $$\theta_{r}=\frac{\sum_{L^{i}\in L^{n}}\arctan\left(a^{i}\right)}{n}$$

The position of the robot between the models $L^{left}$ and $L^{right}$ is defined as

(19) $$x_{r}=\left\{\begin{array}{cc} \frac{|b^{left}|}{|b^{right}|}-1 & \mathrm{if}\;|b^{left}\left|<\right|b^{right}|\\ 1-\frac{|b^{left}|}{|b^{right}|} & \mathrm{otherwise} \end{array},\right.$$

**Figure 9:**
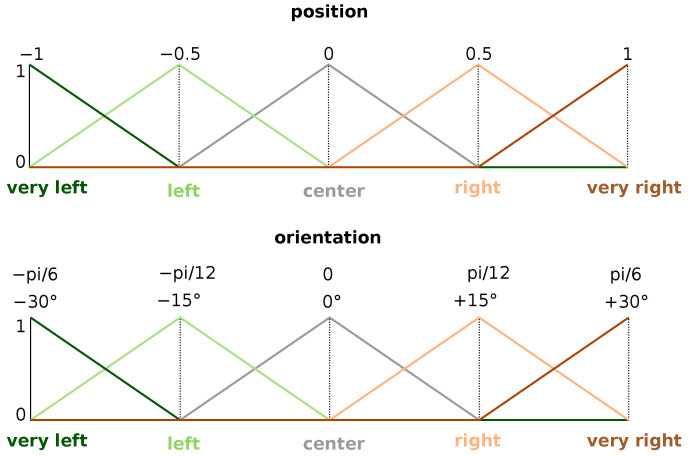
Input membership functions.

These are the inputs of the fuzzy controller ($x_{r}$ and $\theta_{r}$). To proceed to the fuzzification, the membership functions depicted in Figure 9 are considered. For instance, a position of −0.25 will be considered as 0.5 left and 0.5 center.

#### 3.3.2. The Rules

**Figure 10:**
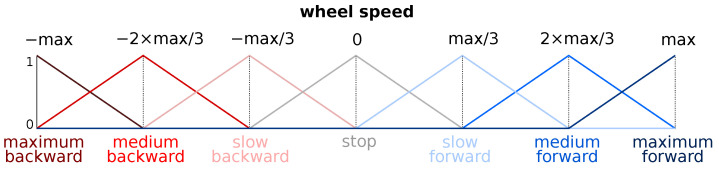
Output membership functions. Note that the left wheel speed and the right wheel speed have the same membership function, and that “max” means the maximal possible speed value.

Before defining the rules, it is necessary to define the fuzzy output membership functions. These functions are the same for the left and the right wheels (note that the considered robot is a two-wheeled differential robot) and are depicted in Figure 10.

**Figure 11:**
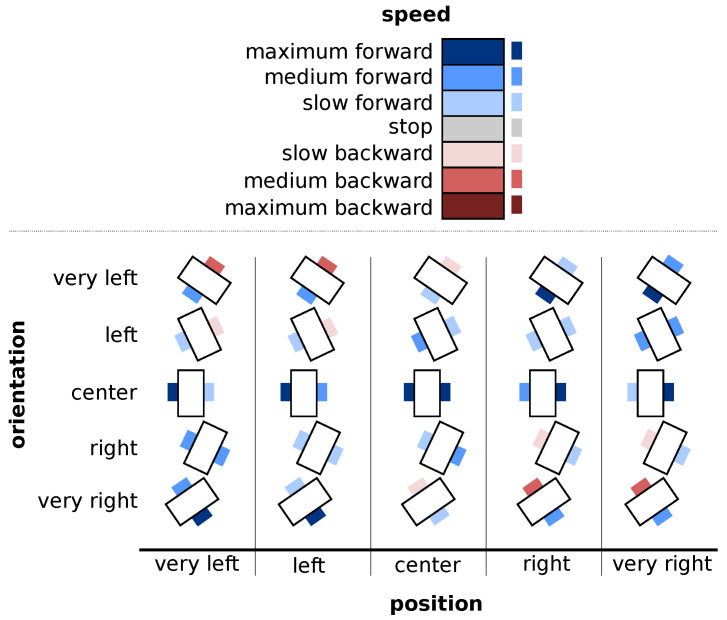
Rule chart. The detailed rules for the left and right wheel speeds according to the position and orientation of the robot.

A fuzzy controller’s last step is to define its rules. These rules aim to keep the robot at the center of two rows, and parallel to them. Figure 11 shows a graphical representation of the defined rules and Table 1 details them.

#### 3.3.3. Operators Summary

The considered operators for the fuzzy controller are as follows:

- AND operator: minimum;
- OR operator: maximum;
- Implication method: Algebraic product;
- Aggregation method: maximum;
- Defuzzification method: the centroid method. This corresponds to the “center of mass” of the results.

## 4. Simulation and Results

To test the algorithms under several controlled environments, a simulation has been designed based on ROS middle-ware and Gazebo robot simulation. These tools are widely used in the robotics community, and thus in agricultural robotics [16,17,18]. It can be noticed that all the source code of this simulation can be downloaded from GitHub [7]. This section presents the developed simulation as well as the methodology used to test the algorithms and the results of the conducted tests.

### 4.1. The Simulation

**Figure 12:**
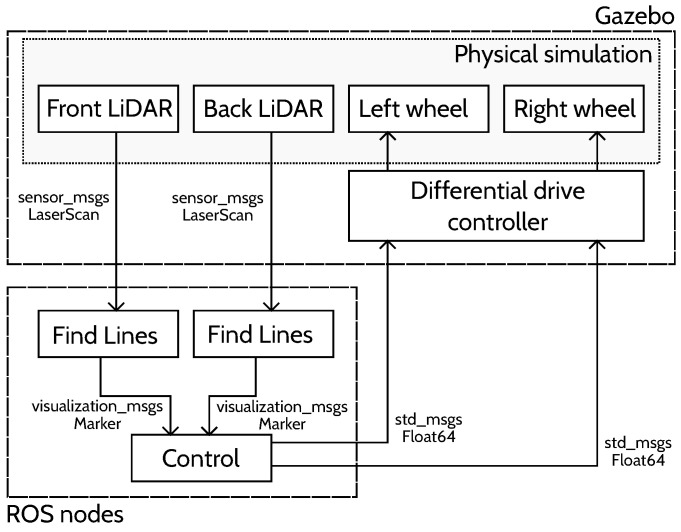
The simulator overview.

Figure 12 presents an overview of the simulation. Gazebo is used to simulate the physics of the system: it generates the LiDAR measurements from the environment and the robot’s pose, and moves the simulated robot according to the wheel speed.

ROS nodes (i.e., programs) were developed to implement the line finding algorithms and the control algorithm.

To handle the fuzzy controller, the open source FuzzyLite C++ library was used [19].

#### 4.1.1. The Robot

**Figure 13:**
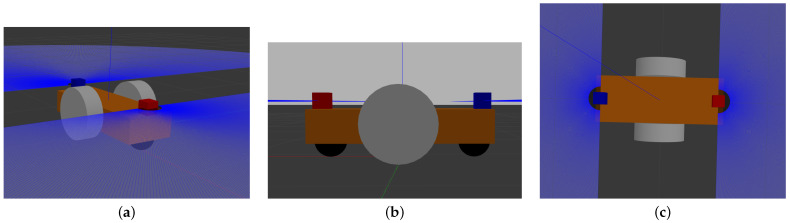
The simulated robot: the white wheels are the differential wheels, the black balls are the caster wheels and the blue/red boxes are the LiDARs. Note that the blue rays depict the LiDAR measurements. (**a**) General view; (**b**) Side view; (**c**) Top view.

The simulated robot is a two-wheeled differential robot, with two caster wheels for stability reasons. Figure 13 depicts the considered robot.

The robot is equipped with two LiDAR sensors, one at the front and one at the back of the robot. Both LiDAR characteristics are:

- Update rate: 40 Hz;
- Number of measurements: 360;
- Minimum angle: $-\frac{\pi }{2}$;
- Maximum angle: $\frac{\pi }{2}$;
- Minimum range: 0.1 m;
- Maximum range: 4 m;
- Resolution: 0.01 m;
- Measurement noise: a Gaussian noise with a 0 mean and a 0.01 standard deviation is considered
  (20) $$f_{noise}\left(x\right)=\frac{1}{0.01\sqrt{2\pi }}e^{-\frac{1}{0.0002}x^{2}}.$$

Two LiDAR sensors are used so that the robot will have the same amount of information from its front as from its back. This symmetry helps to handle the change of row: indeed, the robot still detects the crops when moving out of a row as the *back* LiDAR sensor still detects the plants behind it. Furthermore, the robot does not have to turn around when changing rows, it just has to go *backwards*.

#### 4.1.2. The Simulated Environments

**Figure 14:**
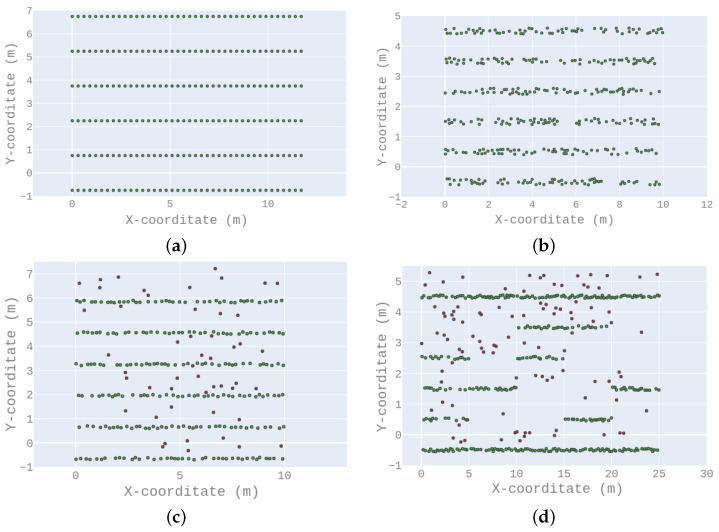
Simulated test crops. The green dots represent crops while the red dots represent weeds. (**a**) Crop 1; (**b**) Crop 2; (**c**) Crop 3; (**d**) Crop 4.

Four fields were designed to test the algorithms. From the *easiest* to the *hardest*, the set-ups are:

- Crop 1, Figure 14a. This corresponds to the easiest configuration, which is a five-row crop, with equal rows, proper plant positioning, and without any weed;
- Crop 2, Figure 14b. In this configuration weeds are still not considered. Nevertheless, the plants are randomly spaced among the rows;
- Crop 3, Figure 14c. This is the first configuration with weeds (depicted as 50 red dots on the Figure 14);
- Crop 4, Figure 14d. This corresponds to the hardest configuration, which is 5 uneven rows with holes and weeds (100 in this case).

It can be noticed that the distances between the rows are not the same for all the simulated crops, but remain the same inside a crop.

### 4.2. Methodology

The following experiments were designed to test two things:

- How the presented refinements affect the line finding results;
- Considering the presented navigation algorithm: if the robot is able to autonomously navigate in the fields and how reliable the navigation is.

To do that, the same control approach (described in Section 3) has been tested with six different line finding algorithms: The two published algorithms Pearl [9] and Ruby [8], and the four proposed Ruby refinements (Section 2.2), which are Ruby Genetic (RG), Ruby Genetic One Point (RGOP), Ruby Genetic One Point Positive/Negative (RGOPPN), and Ruby Genetic One Point Positive Negative Infinity (RGOPPNI).

These six navigation algorithms (a navigation algorithm is the association of a line finding algorithm with the control approach) were tested in the four previously described environments (Figure 14).

**Figure 15:**
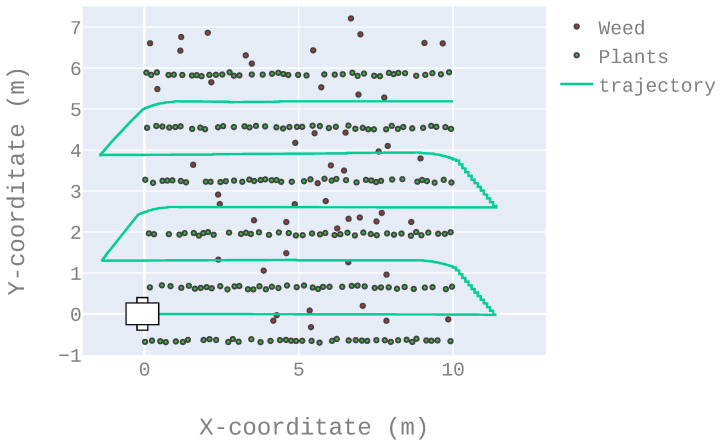
Example of trajectory: the robot starts at the position (0, 0), parallel to the crops, as depicted in the figure. Then it has to reach the end of the fifth row without running over the plants. This figure depicts an example of a successful trajectory.

A test run was done as follows: given a crop and a navigation algorithm, the robot is initially placed at the (0, 0) position of the environment and oriented according to the rows (as shown in Figure 15). Then the robot has to autonomously move through the rows until it reaches the end of the fifth row. Figure 15 presents an example of successful trajectory in the crop 3 environment. Before each run, the robot only has three pieces of information:

- It is at the beginning of the first row between two plant rows;
- It has to navigate through five rows;
- The first new row will be on the left.

Aside from this, it does not know the number of outliers, the distance between the rows, nor the length of the rows.

It can be noticed that for all the runs, the algorithms constants and thresholds do not change, even when changing the field environment. Furthermore, when starting a new run, all the information gathered during the previous run (e.g., the distance *d*), is removed.

The full experiment is presented in Algorithm 5: all the algorithms are tested five times over all the crops.   
**Algorithm 5:** Test plan.
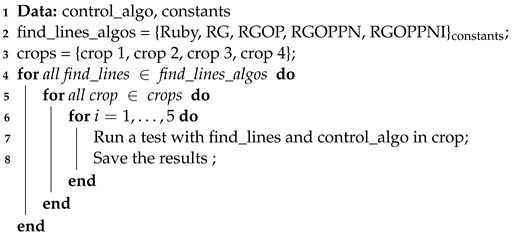


### 4.3. Experiment Results

Several criteria were considered to compare these algorithms.

**Figure 16:**
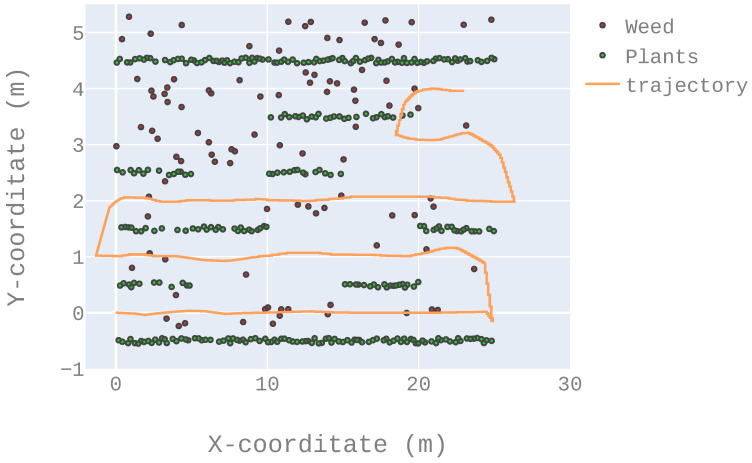
An example of a failed trajectory: the robot reaches the end of the fifth row but runs over the crop plants.

- The ratio of successful runs, computed as the number of successful runs divided by the total number of runs (i.e., 5). A run is considered successful when the robot managed to reach the end of the fifth row without running over the “green” plants, i.e., the crop. Figure 15 presents an example of a successful run while Figure 16 presents an example of a failed run. Table 2 details the results according to this criterion.
- The mean square error when the robot navigated between two rows (not including the changing row maneuver). In a perfect situation, the robot should be exactly equidistant to the direct left and right rows at any time (once again, not including the changing row maneuver). Table 3 details the results according to this criterion.
- At the end of a row, the LiDAR sensor will have fewer points than it would have between two rows (because it detects fewer plants as it is at the end of the crop). That is, in order to have a fair representation of the following criteria, they were obtained over the 500 first iterations only (that is, before reaching the end of the first row).
  – The average execution time of the line finding algorithm. As the control algorithms are the same for all the tested navigation approaches, it is only relevant to test the line finding execution time (the only part that differs from one navigation algorithm to another). Table 4 details the results according to this criterion.
  – The average number of points processed by the line finding algorithms. While some algorithms consider the raw point data set Z, others use the filtered one $Z^{*}$ (Section 2.2.2). That is, the average number of points, presented in Table 5, allows us to verify that for the 500 first iterations the line finding algorithms considered the same number of points (all the Z and all the $Z^{*}$ have consistent sizes).
  – The average execution time per 100 points. Since the line finding algorithm does not consider the same number of points (Z versus $Z^{*}$), the execution time presented in Table 4 may not be comparable regarding only the line finding processes. That is, Table 6 provides an execution time normalized on the processing of 100 points.

Regarding the data presented in the tables, it appears that filtering the input data with a genetic approach (Ruby Genetic One Point) increases the robustness and improves the precision and the computation time. As a matter of fact, combining the Ruby Genetic One Point line finding algorithm with the control algorithm provides a 100% success rate over the five experimental crops (Table 2). It also appears to be the most stable approach (Table 3). However, adding the positive/negative filtering and allowing a point to be associated with several models at the same time results in worse performance than RGOP (Table 2).

The number of processed points is consistent with what is expected: the first algorithms named Ruby and RG consider the same number of points, while the algorithms RGOP, RGOPPN, and RGOPPNI use fewer points (due to the filter point set $Z^{*}$). This can be seen in Table 5.

The considered sensors have a rate of 40 Hz (Section 4.1.1), thus they provide a data set every 25 ms. According to Table 4, the RGOP approach needs less than 3 ms to process a data set. That is, it can be used in real time with this sensor, as can all the other algorithms.

It must be emphasized that the crops (more precisely the weed positions) were randomly generated (the source code for the generation of the simulated crops is available in Ref. [7]). This suggests that this navigation approach will provide reliable results in a wide range of crop configurations. Even if the approach needs to be tested in real conditions, the simulation results are more than encouraging.

## 5. Conclusions

In this paper a novel approach to extract crop rows from LiDAR data is presented. This approach has been coupled with a fuzzy controller to propose a complete navigation algorithm.

A simulator was developed to test this approach in various field situations, and the results are positive: according to the simulated environments, the approach appears to perform well even when the crop has uneven rows with holes and weeds.

Future work will be focused on implementing this approach into an experimental platform and performing navigation in actual fields. Furthermore, cameras will be added to the loop in order to limit the current assumptions about the field. 

## Figures and Tables

**Table 1:** A more classical representation of the controller rules. L: left, R: right, V: very, C: center, 1: slow, 2: medium, 3: maximum, F: forward, B: backward.

**Left Wheel Speed**						
		Position				
		VL	L	C	R	VR
Orientation	VL	2 F	2 F	1 F	3 F	3 F
	L	1 F	1 F	2 F	1 F	2 F
	C	3 F	3 F	3 F	2 F	1 F
	R	2 F	1 F	1 F	1 B	1 B
	VR	2 F	1 F	1 B	2 B	2 B
**Right Wheel Speed**						
		Position				
		VL	L	C	R	VR
Orientation	VL	2 B	2 B	1 B	1 F	2 F
	L	1 B	1 B	1 F	1 F	2 F
	C	1 F	2 F	3 F	3 F	3 F
	R	2 F	1 F	2 F	1 F	1 F
	VR	3 F	3 F	1 F	2 F	2 F

**Table 2:** The ratio of successful runs regarding the total number of tries (five, in this case). A result of 1 means that the algorithm never failed, a result of 0 means that the robot never reached the end of the field without crushing crop plants.

Successful Runs (%)					
	**Crop 1**	**Crop 2**	**Crop 3**	**Crop 4**	**Mean**
**Ruby**	0.8	0.8	1	0.2	0.75
**RG**	0.8	0.8	0.4	0	0.5
**RGOP**	1	1	1	1	1
**RGOPPN**	1	1	1	0.8	0.95
**RGOPPNI**	1	1	1	0	0.75

**Table 3:** The positioning error that the robot made when moving between two crop rows. In a perfect situation, the robot should be exactly in the middle of the two crop rows, and thus should have an error of 0.

Mean Squared Error (mm^2^)					
	**Crop 1**	**Crop 2**	**Crop 3**	**Crop 4**	**Mean**
**Ruby**	2900	360	120	5500	2200
**RG**	8700	3100	89	9640	5300
**RGOP**	65	430	67	750	320
**RGOPPN**	300	700	140	2240	840
**RGOPPNI**	67	310	260	60,000	15,000

**Table 4:** The average execution time for the line finding algorithms (LiDAR data to models) during the 500 first iterations of each run.

Average Execution Time (ms)					
	**Crop 1**	**Crop 2**	**Crop 3**	**Crop 4**	**Mean**
**Ruby**	4.14	21.15	9.39	9.61	11.07
**RG**	5.25	8.12	6.56	6.11	6.51
**RGOP**	2.35	3.44	2.9	2.7	2.84
**RGOPPN**	1.58	3.57	2.8	2.37	2.58
**RGOPPNI**	3.3	6.37	4.42	4.05	4.5

**Table 5:** The average number of points considered by the line finding algorithms during the 500 first iterations of each run.

Average Number of Points					
	**Crop 1**	**Crop 2**	**Crop 3**	**Crop 4**	**Mean**
**Ruby**	91	179.3	122.54	120.36	128.3
**RG**	91.16	178.1	124.53	120	128.4
**RGOP**	40.1	73.12	54.18	47.16	53.64
**RGOPPN**	40	73.15	54.19	47.12	53.61
**RGOPPNI**	40.08	73.96	54.19	47.17	53.85

**Table 6:** The average execution time per 100 points for the line finding algorithms (LiDAR data to models) during the 500 first iterations of each run.

Execution Time /100 Points (ms)					
	**Crop 1**	**Crop 2**	**Crop 3**	**Crop 4**	**Mean**
**Ruby**	4.54	11.79	7.66	7.98	7.99
**RG**	5.75	4.55	5.26	5.09	5.11
**RGOP**	5.86	4.7	5.35	5.7	5.4
**RGOPPN**	3.95	4.88	5.16	5.02	4.75
**RGOPPNI**	8.2	8.61	8.15	8.58	8.38

## Data Availability

The simulator and the experimental environments are available in the Github repository: https://github.com/PolytechAngersMecatroniqueClub/istiaENGRAIS (accessed on 15 November 2022).

## Nomenclature

$p\in R^{2}$	A 2D point (provided by the LiDAR sensor for instance), $p=(x_{p},y_{p})$
N	The set of neighboring points such that an element (p,q)∈N corresponds to two points *p* and *q* in the same neighborhood
$L_{j}$	A model (a line for the crop navigation case), $L_{j}:f\left(x\right)=a_{j}x+b_{j}$
$L^{j}$	The $j^{th}$ filtered model, $L^{j}:f\left(x\right)=a^{j}x+b^{j}$
$L^{left}$	The filtered model closest to the left of the robot, $L^{left}:f\left(x\right)=a^{left}x+b^{left}$
$L^{right}$	The filtered model closest to the right of the robot, $L^{right}:f\left(x\right)=a^{right}x+b^{right}$
$L^{j,-1}$	The $j^{th}$ filtered model of the previous iteration
$L_{\emptyset }$	The empty model (for the outliers)
$L_{i}=\left\{L_{j}\right\}$	A set of models
$L^{-1}$	A set of models from the previous call of line finding
$L^{n}$	A set of *n* filtered models, $L^{n}=\left\{L^{1},L^{2},\ldots ,L^{n}\right\}$
$L^{n,-1}$	The set of *n* filtered models of the previous iteration
L(p)	The model associated with the point *p*
E(L)	The total energy of the model set L
$E_{N}\left(L\right)$	The penalty energy of associating close points to different models
$E_{\emptyset }\left(L\right)$	The outlier energy of the model set L
$E_{L}\left(L_{j}\right)$	The energy of the model $L_{j}$
$P(L_{j})$	The set of points that are associated with the model $L_{j}$, $P\left(L_{j}\right)=\left\{p\in Z|L\left(p\right)=L_{j}\right\}$
Z={p}	The set of all the points
$Z^{*}$	A set of points based on Z, such that all the points that are close enough in Z are fused together
||X||	The Euclidean distance of the *X* expression
$\psi_{j}$	Number of models parallel to model $L_{j}$
ρ,λ,ζ,τ,α,β	Constants, heuristically chosen
$\delta_{\neq }\left(L\left(p\right),L\left(q\right)\right)$	A function that equals 1 if the point *p* is not associated with the same model as the point *q*, 0 otherwise
$w_{pq}$	A weight associated with $\delta_{\neq }\left(L\left(p\right),L\left(q\right)\right)$ in the computation of $E_{N}\left(L\right)$
$\delta_{}\left(L_{j},L_{k}\right)$	A function that equals 1 if the models are parallel, 0 otherwise
$\varphi_{j}$	Fitness criteria for Ruby Genetic
$x_{r}$	The position of the robot between two rows
$\theta_{r}$	The orientation of the robot in the field
*d*	The distance between the rows (constant over the all crop)
$i_{best}$	The index of the previous filter that best match the current expected $i^{th}$ one
$b^{i_{best}}$	The *b* value of the previous filter that best match the current expected $i^{th}$ one
